# The Influence of the Internal Design and Layer Thickness on the Accuracy of 3D-Printed Dental Models

**DOI:** 10.3390/ma18174173

**Published:** 2025-09-05

**Authors:** Jong-Hak Ahn, Jae-Won Choi

**Affiliations:** Department of Dental Laboratory Science, College of Health Science, Catholic University of Pusan, 57 Oryundae-ro, Geumjeong-gu, Busan 46252, Republic of Korea

**Keywords:** 3D printing, dental model, accuracy, internal design, layer thickness

## Abstract

This study investigated the effect of the internal design and layer thickness on 3D-printed dental models. The internal designs were classified based on the presence or absence of the palatal surface (Opened or Closed palate) and the outer wall thickness (1 mm, 3 mm, and Full). They were named as follows: O1, O3, OF, C1, C3, and CF. Based on the internal design and layer thickness (50 and 100 µm), a total of 12 experimental groups were created (n = 120). The control group was fabricated using dental stone (n = 10). The measurement sites were defined as follows: the mesiodistal width (MD) and occlusocervical height (OC) of the crown and the intercanine width (ICW) and intermolar width (IMW). Statistical significance was tested using a two-way ANOVA, one-way ANOVA, and independent sample *t*-test (α = 0.05). In comparisons with and without palatal surfaces, O3 and C3 showed a similar accuracy (*p* > 0.05) in the MD and OC, but C3 showed better (*p* < 0.05) or similar values (*p* > 0.05) than O3 in the ICW and IMW. Meanwhile, there was no significant difference between OF and CF in all measurement sites (*p* > 0.05). In comparisons of the outer wall thickness, CF showed a higher accuracy than C1 at all measurement sites (*p* < 0.05), whereas no statistical significance was observed between CF and C3 except for the ICW (*p* > 0.05). OF and CF did not show statistical significance according to the layer thickness at all measurement sites (*p* > 0.05). In all measurement sites except OC, the experimental group showed a superior (*p* < 0.05) or similar accuracy than the control group (*p* > 0.05). Within the limitations of this study, a 3 mm outer wall, including the palatal surface, and a 50 µm layer thickness is recommended as the optimal 3D printing condition for dental model fabrication.

## 1. Introduction

Dental models are used for diagnosis, treatment planning, patient consultation, and the fabrication of prostheses [1]. Traditional stone models have drawbacks, including long fabrication times; large volumes; and susceptibility to breakage, wear, and deformation [2]. Digital models obtained through intraoral scanners are easy to store, transmit, and analyze and are not limited by physical storage space [3]. Digital impressions using intraoral scanners offer greater patient comfort due to shorter acquisition times and reduce the waste of dental materials [4]. A study by Zarean et al. [5] reported that digital models can serve as alternatives to stone models. However, the margin and inner adaptation of the final prosthesis and the bite and contact areas cannot be directly verified on digital models. Thus, physical models are still required in many clinical situations.

Methods for fabricating physical models based on digital data were largely divided into subtractive manufacturing and additive manufacturing [6]. Subtractive manufacturing enables reproduction and mass production, but it consumes a large amount of material and has limitations in reproducing areas smaller than the milling bur size [7].

Additive manufacturing offers the advantages of minimal material waste and the ability to produce fine details with greater precision [8]. However, compared to subtractive manufacturing, it has its own limitations, such as restricted material options, an inability to print objects larger than the build plate, and the need for post-processing steps like cleaning and light-curing after printing [9]. Meanwhile, previous studies that have compared and evaluated the three-dimensional accuracy of dental models fabricated by subtractive and additive manufacturing reported that models produced by additive manufacturing exhibited higher accuracy [10].

In the dental field, the vat photopolymerization method, in which material in a resin vat is cured layer by layer using a UV light source, is widely used. Representative examples include SLA (Stereolithography Apparatus), DLP (Digital Light Processing), and LCD (Liquid Crystal Display) systems [11,12]. The LCD method selectively blocks light using an LCD screen to photopolymerize the resin in the vat during the printing process [13]. The LCD method is cost-effective and capable of high-resolution output thanks to the LCD screen, but it has the drawback of a short lifespan, requiring regular maintenance [12,13]. A study by comparing the accuracy of dental models produced using SLA, DLP, and LCD technologies reported that the LCD method is sufficiently applicable in clinical settings [14]. Accordingly, this study employed the LCD method to fabricate dental models.

Errors in dental models are closely related to the accuracy and longevity of the final prosthesis [15,16]. Therefore, working models must ensure precision while reproducing the intraoral teeth and surrounding tissues in order to fabricate prostheses with accurate marginal and internal adaptations [17]. Previous studies on the accuracy of 3D-printed models have typically compared them to traditionally fabricated dental models [18]. Some studies have compared them to subtractive manufacturing methods [10], while others have evaluated various factors affecting the accuracy of 3D-printed dental models, including the printer type [3], internal structure and presence of cross plates [19], outer wall thickness [20], layer thickness and print position [21], print orientation [22], and cleaning and post-curing methods [23]. However, no previous studies have simultaneously evaluated the effects of the internal design (palatal plate presence/absence and outer wall thickness) and layer thickness on the accuracy of 3D-printed dental models.

Therefore, the objective of this study is to investigate the effect of various internal designs and layer thicknesses on the accuracy of 3D-printed dental models.

The first null hypothesis states that there is no interaction between the internal design and layer thickness. The second null hypothesis states that the internal design has no effect on the accuracy of 3D-printed dental models. The third null hypothesis states that layer thickness has no effect on the accuracy of 3D-printed dental models.

## 2. Materials and Methods

**Figure 1:**
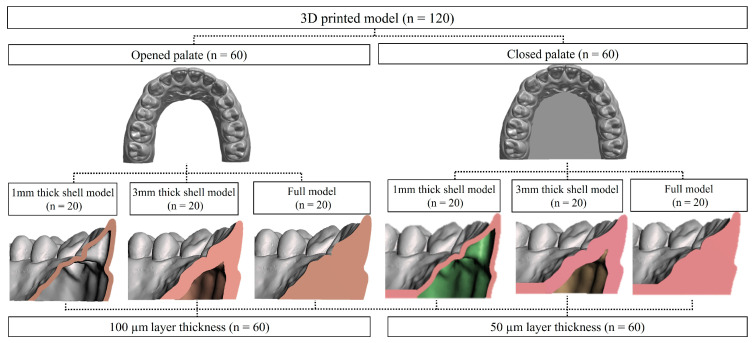
Flowchart of test.

To fabricate the reference model, an impression was taken of a maxillary dentiform (PER5001-UL-SCP-AK-28, Nissin Dental, Kyoto, Japan) using silicone impression material (CharmFlex^®^, Dentkist Ltd., Seongnam-si, Gyeonggi-do, Republic of Korea). The reference model was fabricated using epoxy resin (Exakto-form^®^, Bredent GmbH & Co. KG, Senden, Germany). To produce the control group, impressions were taken ten times from the reference model using silicone material, and dental type IV stone (FUJILOCK^®^ EP, GC, Tokyo, Japan) was used to fabricate ten control models (n = 10). To fabricate the experimental groups, the reference model was scanned using an intraoral scanner (Trios4, 3Shape, Copenhagen, Denmark) to acquire three-dimensional data and create a CAD reference model (CRM). The internal designs were edited using a modeling program (Meshmixer, Autodesk, Mill Valley, CA, USA). The palatal surface was defined as either Opened palate or Closed palate, depending on the presence or absence of a 1.5 mm thick cube-shaped structure. Outer wall thicknesses (1 mm, 3 mm, Full) were applied using the hollow function and exported as STL files. Using slicing software (Halot Box v4.6.0, Creality, Shenzhen, China), the models were divided into 12 groups based on internal design and layer thickness (50 µm, 100 µm), and each was assigned an abbreviation. A total of 120 experimental models were printed (10 models per group) using an LCD 3D printer (Halot Sky, Creality, Shenzhen, China) (Figure 1).

**Figure 2:**
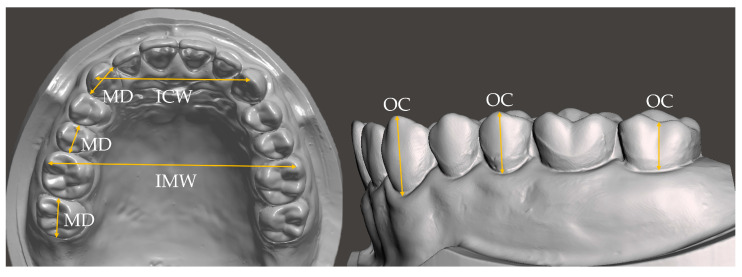
Measured site and device in this study. MD: width from the mesial contact point to the distal contact point; OC: height from the gingival margin to the cusp tip; ICW: intercanine width; and IMW: intermolar width.

After printing, the models were washed for 5 min using a resin washer (Wash&Cure, Anycubic, Shenzhen, China) and then post-cured for 1 min using a light-curing unit (PROBO Cure2, Dio, Busan, Republic of Korea). Eight measurement sites were defined, and measurements were taken using a digital caliper with a tolerance of 0.01 mm (Type 293-561, Mitutoyo, Kawasaki, Japan) [18,21,24]. The measurement sites included the mesiodistal width (MD) and occluso-cervical height (OC) of the maxillary right canine, second premolar, and second molar, as well as the inter-canine width (ICW) and intermolar width (IMW) (Figure 2) [24,25].

Measurements were performed by a single trained researcher, who measured each site thrice. Accuracy was evaluated by calculating the mean and standard deviation of the absolute differences between the reference model and the experimental groups.

The experimental models were scanned using a dental scanner (E3, 3Shape, Copenhagen, Denmark), and STL files were extracted. Using an overlay software (GOM Inspect 2019, GOM GmbH, Braunschweig, Germany), the STL files of the 12 experimental groups were superimposed on the CAD reference model using the Prealignment tool, and a qualitative evaluation was conducted using the Surface Comparison function with a tolerance of ±0.1 mm within a range of ±0.5 mm [26].

For statistical analysis, SPSS software version 29.0 (SPSS Inc., Chicago, IL, USA) was used. A two-way ANOVA was performed to examine interaction effects. Post hoc analysis for interaction effects was conducted using Bonferroni pairwise comparisons. To compare accuracy across measurement sites, one-way ANOVA was used, followed by Tukey’s HSD (Honestly Significant Difference) test, or the Kruskal–Wallis test, with Dunn’s test and Bonferroni correction. For statistical comparison based on layer thickness, either an independent sample *t*-test or the Mann–Whitney U test was performed. Intra-examiner reliability was assessed by calculating the intraclass correlation coefficient (ICC) using a two-way random effects model. The level of statistical significance was set at 0.05.

## 3. Results

**Figure 3:**
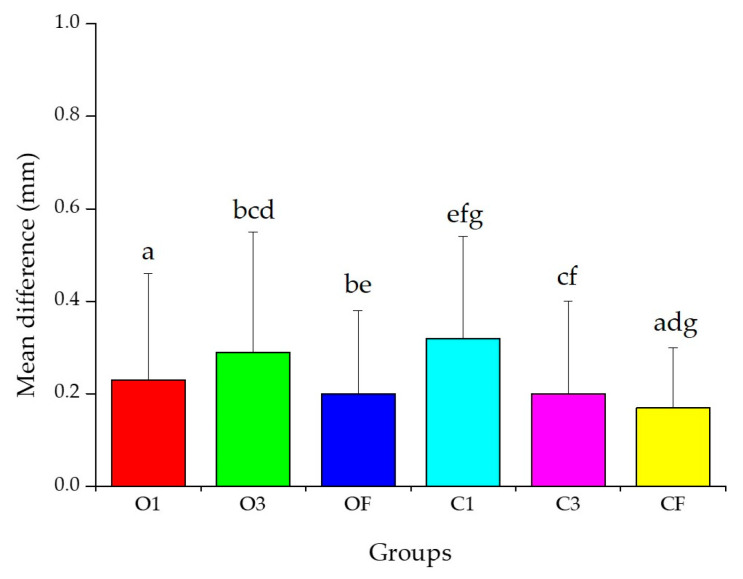
Mean difference from two-way ANOVA for internal design. Same letters indicate significant differences (*p* < 0.05). Data mean and standard deviation values.

**Figure 4:**
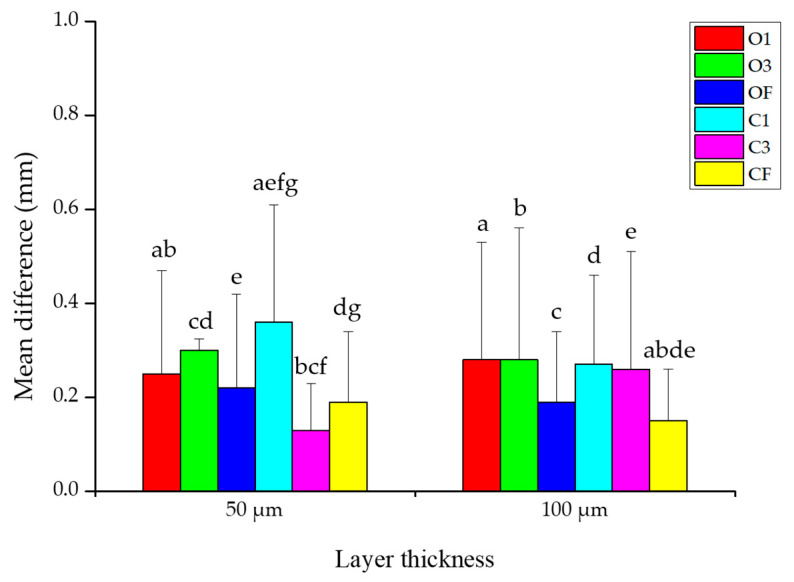
Mean difference in internal design according to layer thickness. Same letters indicate significant differences (*p* < 0.05). Data mean and standard deviation values.

The results of the two-way ANOVA analyzing the effect of the internal design and layer thickness on the accuracy of 3D-printed dental models are presented (Table 1, Figure 3 and Figure 4). Main effect tests were conducted to evaluate the independent effects of internal design types and two-layer thicknesses on the accuracy. The effect of the internal design on accuracy was statistically significant (F = 11.438, *p* < 0.001). However, the effect of the layer thickness on accuracy was not statistically significant (F = 0.081, *p* = 0.776) (Table 1). A significant interaction effect between the internal design and layer thickness was observed (F = 4.845, *p* < 0.001) (Table 1, Figure 4).

In the main effect test for the internal design, the CF group showed a significantly higher accuracy than all other groups except for OF and C3 (*p* < 0.05) (Figure 3).

From the interaction analysis, at a layer thickness of 50 µm, the C3 group showed the highest accuracy (*p* < 0.05), although the differences compared to OF and CF were not statistically significant (*p* > 0.05). At 100 µm, CF showed the highest accuracy (*p* < 0.05), and the difference with OF was not statistically significant (*p* > 0.05) (Figure 4).

To evaluate the intra-examiner reliability for measurements of both control and experimental groups, ICCs were calculated. The average ICC ranged from 0.806 to 0.999 for the control group and from 0.770 to 0.999 for the experimental groups (Table 2).

In the comparison based on the palatal surface presence, the O1 group demonstrated an equal (*p* > 0.05) or higher accuracy (*p* < 0.05) than the C1 group across all measurement sites, with no significant difference observed between OF and CF (*p* > 0.05). In the comparison between O3 and C3, no significant differences were found in MD and OC (*p* > 0.05). C3 showed either a superior (*p* < 0.05) or comparable accuracy (*p* > 0.05) in inter-arch distances (ICW and IMW) (Table 3).

In the comparison based on the outer wall thickness, OF showed either an equal (*p* < 0.05) or higher accuracy (*p* > 0.05) than O3 across all measurement sites, with no significant differences between O1 and OF except for the IMW (*p* > 0.05). CF exhibited a significantly higher accuracy than C1 across all measurement sites (*p* < 0.05). There was no statistically significant difference between CF and C3 except for the ICW (*p* > 0.05) (Table 3).

Regarding the effect of the layer thickness, there was no significant difference in accuracy based on the layer thickness across all groups except C3 for MD and OC (*p* > 0.05). In the ICW and IMW, OF and CF did not show statistically significant differences based on the layer thickness (*p* > 0.05). For all measurement sites, the 50 µm layer thickness showed a higher (*p* < 0.05) or comparable accuracy (*p* > 0.05) compared to 100 µm in O1, O3, and C3, while in C1, the 100 µm thickness showed a higher (*p* < 0.05) or similar accuracy (*p* > 0.05) compared to 50 µm (Table 3).

In comparison to the control group, the experimental groups fabricated by 3D printing showed an equal (*p* > 0.05) or superior accuracy (*p* < 0.05) in all measurement sites except for OC (Table 3).

**Figure 5:**
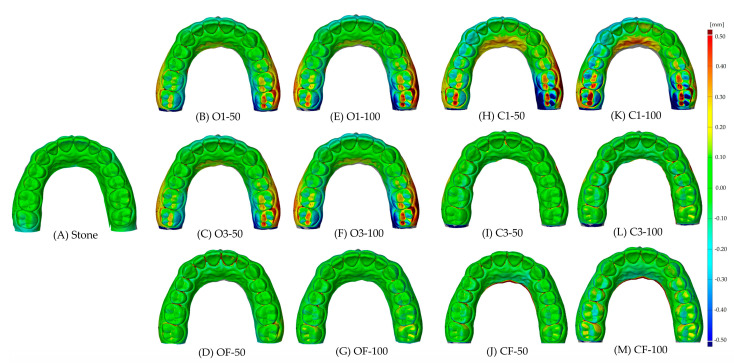
Color map according to internal design and layer thickness compared to stone.

In the qualitative analysis, the deviation was evaluated within a ±0.5 mm range using a ±0.1 mm tolerance (Figure 5). Regardless of the palatal surface presence, greater deviation was observed in the posterior region than in the anterior region when the outer wall thickness was 1 mm (Figure 5B,E,H,K). At an outer wall thickness of 3 mm, less deviation was observed in the Closed palate groups compared to the Opened palate groups, with a positive deviation noted on the buccal side and a negative deviation on the palatal side (Figure 5C,F). Overall, smaller deviation values were observed in OF, C3, and CF compared to O1, O3, and C1 (Figure 5).

## 4. Discussion

The accuracy and durability of 3D-printed dental models are influenced by various factors, such as the outer wall thickness, layer thickness, printing angle, and post-curing time [3,20,21,22,23]. In this study, the effects of the internal design (presence or absence of the palatal surface and outer wall thickness) and layer thickness on the accuracy of 3D-printed dental models were investigated to identify optimal conditions that provide high accuracy while minimizing the material consumption and printing time for potential clinical applications. The first null hypothesis, that there is no interaction between the internal design and layer thickness, was rejected by the two-way ANOVA. The second null hypothesis, that the internal design has no effect on the accuracy of 3D-printed dental models, was also rejected. The third null hypothesis, that the layer thickness does not affect the accuracy of the 3D-printed dental model, was accepted through the two-way ANOVA.

According to previous studies, methods for evaluating the accuracy of dental models can be broadly categorized into analog linear measurement methods, in which an examiner uses tools such as a caliper to measure the distance between two reference points on a physical model [25], and digital measurement methods using specialized software [19,25]. Digital methods include linear measurements; a 2D cross-sectional analysis [27]; and a 3D best-fit analysis [20].

A digital measurement requires creating a digital model using a dental scanner. However, the accuracy of digital models can be affected by the user’s proficiency and may be distorted depending on factors such as the scanning angle, resolution, reflectivity, temperature, and humidity [28,29]. Moreover, digital methods can introduce substantial errors during the overlay process, depending on the software’s performance [16]. In studies comparing the accuracy of different types of 3D printers using both calipers and software-based linear measurements, the values measured via software typically showed greater standard deviations [15]. Therefore, in this study, we used a digital caliper, a precision analog measuring tool capable of measuring fine details from 0.1 mm to 0.001 mm. This has been widely used in recent studies as a reliable method, replacing digital measurement methods that utilize error-prone software during scanning and superimposition [19,24,30,31,32,33].

On the other hand, positioning the digital caliper tips at pre-defined measurement points can be challenging for the examiner [30]. Therefore, to minimize errors, a single operator performed three repeated measurements for each site using the same method, and the average value was calculated based on the absolute difference from the reference model. Intra-examiner reliability was assessed by calculating the ICC for each measurement set. According to Koo et al. [34], ICC values within a 95% confidence interval can be classified into four levels of reliability: less than 0.5 indicates poor reliability, 0.5 to 0.75 indicates moderate reliability, 0.75 to 0.9 indicates good reliability, and greater than 0.9 indicates excellent reliability. In this study, all ICC values were above 0.75, indicating good to excellent reliability. For the MD and OC of the crown, ICC values showed excellent reliability with a minimum value of 0.996. For the ICW and IMW, ICC values demonstrated good reliability with minimum values above 0.770. In a study by Liang et al. [35], measurements of dental models were taken using both digital calipers and software. Consequently, comparisons by measurement region showed that ICC values were lower for arch width measurements than for crown dimensions, consistent with the results of this study. Therefore, the lower ICC values observed for the ICW and IMW compared to the MD and OC suggest that the length of the measurement may influence reliability outcomes.

Previous studies have reported a wide range of clinically acceptable errors for the accuracy of 3D-printed dental models, ranging from under 100 µm to 500 µm [36]. Among them, studies using digital calipers (analog measurement method) reported that the error in the MD and OC was less than 500 µm [21,24,31,37,38], while for the ICW and IMW, the acceptable threshold was often defined as less than a 5% difference from the reference model [25,39]. In this study, all measurements across all sites were within the clinically acceptable margin of error.

In the accuracy analysis based on the presence of a palatal surface, models with a Closed palate showed superior accuracy compared to those with an Opened palate. This finding is consistent with previous studies, such as Shin et al. [19], which reported lower accuracy in 3D-printed dental models when the palatal surface was absent. For inter-arch distances (ICW and IMW), models with a Closed palate also demonstrated a comparable or better accuracy than those with an Opened palate. In the qualitative analysis, less deviation was observed in C3 compared to O3.

In the comparison of accuracy according to the outer wall thickness, results from the two-way ANOVA revealed that models with thicker outer walls (CF, OF, C3) exhibited a significantly higher accuracy. Interestingly, for the ICW measurement, C3 showed a higher accuracy than CF, whereas for all other measurement points, there was no statistically significant difference between C3 and CF. Qualitative evaluation also revealed a comparable accuracy between C3 and CF. In a study by Shin et al. [19], it was reported that during post-curing shrinkage, thicker models resisted deformation more effectively than thinner models. Similarly, a previous study compared the accuracy of 3D-printed dental models with outer wall thicknesses of 1, 2, 3, and 4 mm, as well as fully filled interiors, and found that models with 1 mm walls had the lowest accuracy, whereas the accuracy improved with an increasing wall thickness [20]. Another study by Shin et al. [3] found that as the wall thickness decreased, the palatal shrinkage increased relative to the labial and buccal surfaces, resulting in reduced overall dimensional stability.

Regarding the layer thickness, independent sample *t*-tests revealed significant differences in accuracy based on the layer thickness in O1, O3, C1, and C3 groups, while no significant difference was observed in OF and CF. A study by Sherman et al. [21] comparing models with a 1.5 mm outer and basal wall thickness to fully filled models found that when outer walls were Full, there was no statistically significant difference in the accuracy between 50 µm and 100 µm layer thicknesses, aligning with the findings of the present study. These results suggest that when the outer wall is sufficiently thick, the wall thickness may have a greater impact on accuracy than the layer thickness.

Compared to the control group, the one-way ANOVA revealed that the experimental groups exhibited a comparable or superior accuracy in all measurement areas except for OC, which is related to the tooth height along the Z-axis. This corresponds with findings from Keating et al. [40], which reported that 3D-printed dental models tend to show greater inaccuracies along the Z-axis (related to tooth height) than along the X–Y axes.

This study has some limitations. Since only the internal design and layer thickness were considered when evaluating factors affecting the accuracy of 3D-printed models, the generalizability of the results may be limited. Future research should explore the effects of other variables, such as the printing angle, the object placement on the build plate, the post-curing time and temperature, various light sources and wavelengths during post-curing, the material type, the washing time, and comparisons with other types of 3D printing technologies. Such follow-up studies are expected to provide clearer clinical guidelines for the fabrication of dental models using 3D printing.

## 5. Conclusions

Within the limitations of this study, all experimental groups showed errors within clinically acceptable ranges—less than 500 µm in the crown height and width and less than 5% in inter-arch distances. The 3D-printed dental models fabricated with a 3 mm outer wall, including the palatal surface and a layer thickness of 50 µm, are recommended as optimal printing conditions, offering both high accuracy and efficiency.

## Figures and Tables

**Table 1:** Result from two-way ANOVA for internal design and layer thickness.

Source	Sum of Squares	Df	Mean Square	F	*p*
Corrected model	3.446 ^a^	11	0.313	7.409	<0.001
Intercept	47.968	1	47.968	1134.448	<0.001
Internal design	2.418	5	0.484	11.438	<0.001
Layer thickness	0.003	1	0.003	0.081	0.776
Internal design * Layer thickness	1.024	5	0.205	4.845	<0.001
Error	24.770	948	0.026		
Total	69.230	960			
Corrected total	29.249	959			

a. R Squared = 0.090(Adjusted R Squared = 0.078).

**Table 2:** Intra-examiner ICC values based on absolute agreement between 3D-printed model and stone model according to internal design and layer thickness.

Measurement Point	Layer Thickness	ICC (95% CI)						
		Stone	O1	O3	OF	C1	C3	CF
MD	50	0.998 (0.997–0.999)	0.996 (0.993–0.998)	0.999 (0.997–0.999)	0.997 (0.995–0.999)	0.998 (0.996–0.999)	0.998 (0.997–0.999)	0.998 (0.996–0.999)
	100		0.995 (0.991–0.998)	0.998 (0.995–0.999)	0.997 (0.994–0.998)	0.995 (0.991–0.998)	0.998 (0.994–0.999)	0.998 (0.995–0.999)
OC	50	0.999 (0.998–1.000)	0.999 (0.998–0.999)	0.999 (0.999–1.000)	0.999 (0.999–1.000)	0.999 (0.999–1.000)	0.999 (0.996–1.000)	0.999 (0.998–0.999)
	100		0.998 (0.997–0.999)	0.999 (0.997–1.000)	0.999 (0.998–0.999)	0.998 (0.997–0.999)	0.999 (0.999–1.000)	0.999 (0.998–0.999)
ICW	50	0.885 (0.158–0.990)	0.774 (0.233–0.968)	0.770 (0.319–0.982)	0.880 (0.469–0.986)	0.939 (0.397–0.993)	0.772 (0.372–0.990)	0.891 (0.446–0.988)
	100		0.794 (0.390–0.953)	0.861 (0.474–0.992)	0.797 (0.185–0.976)	0.796 (0.124–0.976)	0.835 (0.240–0.951)	0.794 (0.140–0.978)
IMW	50	0.806 (0.149–0.979)	0.795 (0.435–0.944)	0.890 (0.331–0.770)	0.990 (0.418–0.942)	0.789 (0.034–0.964)	0.792 (0.315–0.937)	0.909 (0.604–0.990)
	100		0.854 (0.589–0.960)	0.793 (0.311–0.973)	0.819 (0.261–0.979)	0.770 (0.096–0.973)	0.865 (0.349–0.884)	0.826 (0.055–0.981)

ICC: Intraclass correlation coefficient; CI: 95% confidence interval.

**Table 3:** Mean difference (mm) and standard deviation (SD) for all measurement points of 3D-printed models and stone models according to internal design and layer thickness.

Measurement Point	Layer Thickness	Groups, Mean Difference (SD)							
		Stone	O1	O3	OF	C1	C3	CF	*p*-Value
MD	50	0.12 ^a^ (0.04)	0.19 ^b^ (0.13)	0.15 ^c^ (0.19)	0.11 ^d^ (0.07)	0.20 ^de^ (0.15)	0.13 ^f^ (0.09)	0.07 ^be^ (0.07)	<0.001
	100	0.12 ^a^ (0.04)	0.13 ^b^ (0.12)	0.18 ^cd^ (0.07)	0.09 ^ce^ (0.06)	0.17 ^ef^ (0.12)	0.13 ^g^ (0.06)	0.07 ^df^ (0.06)	<0.001
	*p*		0.074	0.476	0.238	0.425	0.846	0.801	
OC	50	0.13 ^abcd^ (0.13)	0.19 ^e^ (0.10)	0.26 ^a^ (0.11)	0.19 ^f^ (0.10)	0.29 ^be^ (0.17)	0.27 ^c^ (0.12)	0.24 ^d^ (0.13)	<0.001
	100	0.13 ^ab^ (0.13)	0.19 ^c^ (0.16)	0.24 ^a^ (0.14)	0.17 ^d^ (0.10)	0.24 ^b^ (0.11)	0.16 ^e^ (0.08)	0.18 ^f^ (0.10)	0.003
	*p*		0.867	0.401	0.439	0.254	<0.001	0.069	
ICW	50	0.21 ^a^ (0.06)	0.16 ^b^ (0.10)	0.37 ^cd^ (0.02)	0.17 ^e^ (0.13)	0.53 ^befg^ (0.24)	0.02 ^acf^ (0.01)	0.14 ^dg^ (0.12)	<0.001
	100	0.21 ^a^ (0.06)	0.21 ^b^ (0.16)	0.47 ^abcde^ (0.03)	0.15 ^c^ (0.11)	0.23 ^d^ (0.12)	0.27 ^f^ (0.01)	0.09 ^ef^ (0.06)	<0.001
	*p*		0.440	<0.001	0.912	0.002	<0.001	0.230	
IMW	50	0.51 ^a^ (0.12)	0.23 ^b^ (0.16)	0.78 ^bcde^ (0.02)	0.17 ^c^ (0.09)	0.52 ^f^ (0.17)	0.04 ^adf^ (0.02)	0.19 ^e^ (0.13)	<0.001
	100	0.51 ^ab^ (0.12)	0.60 ^cde^ (0.28)	0.85 ^fghi^ (0.02)	0.16 ^cf^ (0.12)	0.32 ^g^ (0.18)	0.09 ^adh^ (0.02)	0.15 ^bei^ (0.13)	<0.001
	*p*		0.002	<0.001	0.876	0.022	<0.001	0.505	

Values with the same letter are statistically different from each other. MD: mesiodistal; OC: occlusocervical; ICW: intercanine width; IMW: intermolar width.

## Data Availability

The original contributions presented in this study are included in the article. Further inquiries can be directed to the corresponding author.

## References

[1] Jin G., Shin S.H., Shim J.S., Lee K.W., Kim J.E. (2022). Accuracy of 3D printed models and implant-analog positions according to the implant-analog-holder offset, inner structure, and printing layer thickness: An in-vitro study. J. Dent..

[2] Patzelt S.B.M., Bishti S., Stampf S., Att W. (2014). Accuracy of computer aided design/computer-aided manufacturing-generated dental casts based on intraoral scanner data. J. Am. Dent. Assoc..

[3] Rungrojwittayakul O., Kan J.Y., Shiozaki K., Swamidass R.S., Goodacre B.J., Goodacre C.J., Lozada J.M. (2020). Accuracy of 3D printed models created by two technologies of printers with different designs of model base. J. Prosthodont..

[4] Michelinakis G., Apostolakis D., Tsagarakis A., Kourakis G., Pavlakis E. (2020). A comparison of accuracy of 3 intraoral scanners: A single-blinded in vitro study. J. Prosthet. Dent..

[5] Zarean P., Zarean P., Sendi P., Neuhaus K.W. (2023). Advances in the Manufacturing Process of Space Maintainers in Pediatric Dentistry: A Systematic Review from Traditional Methods to 3D-Printing. Appl. Sci..

[6] Paris H., Mokhtarian H., Coatanéa E., Museau M., Ituarte I.F. (2016). Comparative environmental impacts of additive and subtractive manufacturing technologies. CIRP Ann..

[7] Moon J.M., Jeong C.S., Lee H.J., Bae J.M., Choi E.J., Kim S.T., Park Y.B., Oh S.H. (2022). A Comparative Study of Additive and Subtractive Manufacturing Techniques for a Zirconia Dental Product: An Analysis of the Manufacturing Accuracy and the Bond Strength of Porcelain to Zirconia. Materials.

[8] Jayawardane H., Davies I.J., Gamage J.R., John M., Biswas W.K. (2023). Sustainability perspectives—A review of additive and subtractive manufacturing. Sustain. Manuf. Serv. Econ..

[9] Rasheed R.K., Mansoor N.S., Mohammed N.H., Qasim S.S.B. (2023). Subtractive and Additive Technologies in Fixed Dental Restoration: A Systematic Review. J. Tech..

[10] Jeong Y.G., Lee W.S., Lee K.B. (2018). Accuracy evaluation of dental models manufactured by CAD/CAM milling method and 3D printing method. J. Adv. Prosthodont..

[11] Andjela L., Abdurahmanovich V.M., Vladimirovna S.N., Mikhailovna S.I., Yurievich D.D., Alekseevna M.Y. (2022). A review on Vat Photopolymerization 3D-printing processes for dental application. Dent. Mater..

[12] Caussin E., Moussally C., Goff S.L., Fasham T., Troizier-Cheyne M., Tapie L., Dursun E., Attal J.P., François P. (2024). Vat Photo-polymerization 3D Printing in Dentistry: A Comprehensive Review of Actual Popular Technologies. Materials.

[13] Quan H., Zhang T., Xu H., Luo S., Nie J., Zhu X. (2020). Photo-curing 3D printing technique and its challenges. Bioact. Mater..

[14] Revilla-León M., Sadeghpour M., Özcan M. (2020). An update on applications of 3D printing technologies used for processing polymers used in implant dentistry. Odontology.

[15] Kim J.H., Pinhata-Baptista O.H., Ayres A.P., Silva R.L.B., Lima J.F., Urbanoo G.S., No-Cortes J., Vasques M.T., Cortes A.R.G. (2022). Accuracy Comparison among 3D-Printing Technologies to Produce Dental Models. Appl. Sci..

[16] Ender A., Mehl A. (2013). Accuracy of complete-arch dental impressions: A new method of measuring trueness and precision. J. Prosthet. Dent..

[17] Choi J.W., Ahn J.J., Son K., Huh J.B. (2019). Three-Dimensional Evaluation on Accuracy of Conventional and Milled Gypsum Models and 3D Printed Photopolymer Models. Materials.

[18] Kardach H., Szponar-Zurowska A., Biedziak B. (2023). A Comparison of Teeth Measurements on Plaster and Digital Models. J. Clin. Med..

[19] Shin S.H., Lim J.H., Kang Y.J., Kim J.H., Shim J.S., Kim J.E. (2020). Evaluation of the 3D printing accuracy of a dental model according to its internal structure and cross-arch plate design: An in vitro study. Materials.

[20] Shin S.H., Kwon J.S., Shim J.S., Kim J.E. (2021). Evaluating the three-dimensional printing accuracy of partial-arch models according to outer wall thickness: An in vitro study. Materials.

[21] Sherman S.L., Kadioglu O., Currier G.F., Kierl J.P., Li J. (2020). Accuracy of digital light processing printing of 3-dimensional dental models. Am. J. Orthod. Dentofac. Orthop..

[22] Jin M.C., Yoon H.I., Yeom I.S., Kim S.H., Han J.S. (2020). The effect of build angle on the tissue surface adaptation of maxillary and mandibular complete denture bases manufactured by digital light processing. J. Prosthet. Dent..

[23] Unkovskiy A., Bui P.H., Schille C., Geis-Gerstorfer J., Huettig F., Spintzyk S. (2018). Objects build orientation, positioning, and curing influence dimensional accuracy and flexural properties of stereolithographically printed resin. Dent. Mater..

[24] Ellakany P., Al-Harbi F., Tantawi M.E.I., Mohsen C. (2022). Evaluation of the accuracy of digital and 3D-printed casts compared with conventional stone casts. J. Prosthet. Dent..

[25] Hassan W.N.W., Yusoff Y., Mardi N.A. (2022). Comparison of reconstructed rapid prototyping models produced by 3-dimensional printing and conventional stone models with different degrees of crowding. Am. J. Orthod. Dentofac. Orthop..

[26] Arnold C., Rib L., Hey J., Schweyen R. (2024). Dimensional Accuracy of Different Three-Dimensional Printing Models as a Function of Varying the Printing Parameters. Materials.

[27] Maeng J., Lim Y.J., Kim B., Kim M.J., Kwon H.B. (2019). A New Approach to Accuracy Evaluation of Single-Tooth Abutment Using Two-Dimensional Analysis in Two Intraoral Scanners. Int. J. Environ. Res. Public Health.

[28] An H., Langas E.E., Gill A.S. (2024). Effect of scanning speed, scanning pattern, and tip size on the accuracy of intraoral digital scans. J. Prosthet. Dent..

[29] Park M.E., Shin S.Y. (2018). Three-dimensional comparative study on the accuracy and reproducibility of dental casts fabricated by 3D printers. J. Prosthet. Dent..

[30] Hazeveld A., Slater J.J., Ren Y. (2014). Accuracy and reproducibility of dental replica models reconstructed by different rapid proto-typing techniques. Am. J. Orthod. Dentofac. Orthop..

[31] Brown G.B., Currier G.F., Kadioglu O., Kierl J.P. (2018). Accuracy of 3-dimensional printed dental models reconstructed from digital intraoral impressions. Am. J. Orthod. Dentofac. Orthop..

[32] Tsolakis I.A., Papaioannou W., Papadopoulou E., Dalampira M., Tsolakis A.I. (2022). Comparison in terms of accuracy between DLP and LCD printing technology for dental model printing. Dent. J..

[33] Curinga M.R.S., Sousa L.C., Pereira A.L.C., Melo-Segundo H.V., Dantas L.M.C.M., Carreiro A.D.F.P. (2023). Accuracy of models of partially edentulous arches obtained by three-dimensional printing: An in vitro study. J. Indian Prosthodont. Soc..

[34] Koo T.K., Li M.Y. (2016). A Guideline of Selecting and Reporting Intraclass Correlation Coefficients for Reliability Research. J. Chiropr. Med..

[35] Liang Y.M., Rutchakitprakarn L., Kuang S.H., Wu T.Y. (2018). Comparing the reliability and accuracy of clinical measurements using plaster model and the digital model system based on crowding severity. J. Chin. Med. Assoc..

[36] Etemad-Shahidi Y., Qallandar O.B., Evenden J., Alifui-Segbaya F., Ahmed K.E. (2020). Accuracy of 3-Dimensionally Printed Full-Arch Dental Models: A Systematic Review. J. Clin. Med..

[37] Dietrich C.A., Ender A., Baumgartner S., Mehl A. (2017). A validation study of reconstructed rapid prototyping models produced by two technologies. Angle Orthod..

[38] Aly P., Mohsen C. (2020). Comparison of the Accuracy of Three-Dimensional Printed Casts, Digital, and Conventional Casts: An In Vitro Study. Eur. J. Dent..

[39] Czarnota J., Hey J., Fuhrmann R. (2016). Measurements using orthodontic analysis software on digital models obtained by 3D scans of plaster casts: Intrarater reliability and validity. J. Orofac. Orthop..

[40] Keating A.P., Knox J., Bibb R., Zhurov A.I. (2008). A comparison of plaster, digital and reconstructed study model accuracy. J. Orthod..

